# Telomere–metabolism–immunity axis in sarcoma: Immune evasion mechanisms and therapeutic strategies

**DOI:** 10.1002/ctm2.70504

**Published:** 2025-10-20

**Authors:** Ji‐Yong Sung, June Hyuk Kim

**Affiliations:** ^1^ Department of Biological Sciences College of Natural Sciences Seoul National University Seoul South Korea; ^2^ Department of Neurosurgery Seoul National University Bundang Hospital Seoul National University College of Medicine Seongnam‐si South Korea; ^3^ Department of Orthopaedic Surgery National Cancer Center Goyang‐si South Korea

**Keywords:** combinatorial strategy, immune evasion, immunotherapy, metabolic reprogramming, sarcoma, telomere maintenance mechanisms (TMMs)

## Abstract

Sarcomas are a heterogeneous group of mesenchymal malignancies with poor prognosis and limited response to standard therapies, including immune checkpoint inhibitors (ICIs). Tumour‐intrinsic factors—such as telomere maintenance mechanisms (TMMs) and metabolic reprogramming—play central roles in driving immune evasion and therapeutic resistance. Telomerase activation and alternative lengthening of telomeres sustain replicative immortality while influencing the tumour immune microenvironment. In parallel, metabolic adaptations, including glutamine dependency and arginine auxotrophy, further suppress antitumour immunity. Together, TMMs and metabolism form an integrated axis that shapes immune modulation and treatment outcomes. Recent advances—ranging from telomerase‐based vaccines and TMM‐targeted immunotherapies to metabolic modulators combined with ICIs—demonstrate the translational promise of targeting this axis. This review synthesises current knowledge on telomere‒metabolism crosstalk in sarcomas, highlights its impact on immunotherapy response, and outlines future directions for biomarker‐driven, combinatorial strategies to overcome resistance and improve patient outcomes.

**Key points:**

- Telomere maintenance mechanisms (telomerase reverse transcriptase and alternative lengthening of telomeres) reprogram metabolism and dampen innate immune sensing in sarcomas.
- Metabolic rewiring (glutamine addiction, glycolysis and fatty acid oxidation) fosters T‐cell dysfunction and myeloid‐derived suppressor cell accumulation.
- Targeting the telomere‒metabolism‒immunity axis offers strategies to overcome immunotherapy resistance.

## Graphical abstract

1. Telomere maintenance mechanisms (telomerase reverse transcriptase and alternative lengthening of telomeres) reprogram metabolism and dampen innate immune sensing in sarcomas.
2. Metabolic rewiring (glutamine addiction, glycolysis and fatty acid oxidation) fosters T‐cell dysfunction and myeloid‐derived suppressor cell accumulation.
3. Targeting the telomere‒metabolism‒immunity axis offers strategies to overcome immunotherapy resistance.

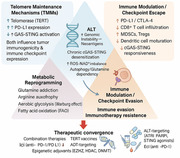

## 1 INTRODUCTION

**FIGURE 1:**
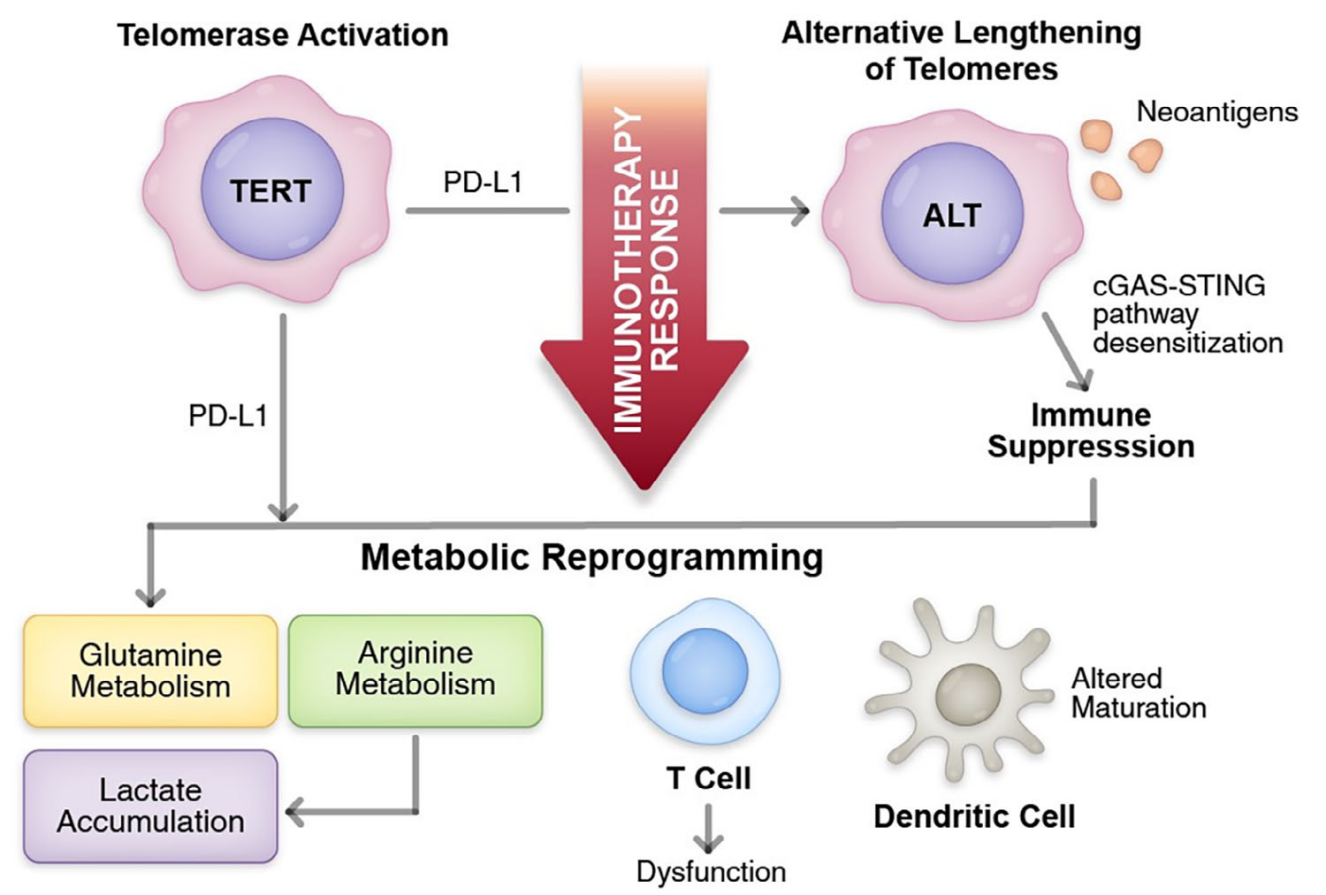
Telomere maintenance mechanisms and metabolic reprogramming cooperatively shape immunotherapy response in sarcomas. The schematic illustrates how distinct telomere maintenance mechanisms (TMMs)—telomerase activation and alternative lengthening of telomeres (ALT)—influence immunotherapy response through metabolic and immune modulation in sarcoma cells. On the left, telomerase activation (TERT) promotes Programmed Death‐Ligand 1 (PD‐L1) expression, contributing to immune suppression and influencing metabolic reprogramming, including alterations in glutamine and arginine metabolism, as well as lactate accumulation. These metabolic changes impair T‐cell function and dendritic cell maturation, further dampening antitumour immunity. On the right, ALT generates neoantigens but simultaneously leads to desensitisation of the cyclic GMP‒AMP synthase (cGAS)‒STING pathway, resulting in immune suppression. Both TMMs converge on metabolic reprogramming, ultimately causing T‐cell dysfunction and altered dendritic cell maturation, which collectively impair effective immunotherapy response. The red downward arrow indicates the net suppression of immunotherapy efficacy driven by these processes.

Sarcomas are a diverse group of mesenchymal malignancies encompassing more than 70 histological subtypes, broadly classified into soft tissue sarcomas and bone sarcomas. Despite their relative rarity, sarcomas disproportionately affect children, adolescents and young adults, and are often associated with high‐grade pathology, early metastasis and poor prognosis. Current treatment strategies—including surgical resection, radiation and cytotoxic chemotherapy—are frequently ineffective in advanced or metastatic cases, necessitating novel therapeutic approaches. Recent epidemiological data highlight that although sarcomas represent only ∼1% of adult malignancies, they account for up to 15%‒20% of paediatric solid tumours, with 5‐year survival rates for advanced disease remaining below 20% in both Surveillance, Epidemiology, and End Results Program and European population‐based datasets. These figures highlight the pressing clinical demand for innovative treatment strategies Figure 1.

In recent years, immune checkpoint inhibitors (ICIs), particularly those targeting Programmed Cell Death Protein‐1 (PD‐1)/Programmed Death‐Ligand 1 (PD‐L1) and Cytotoxic T‐Lymphocyte Antigen 4 (CTLA‐4), have revolutionised cancer therapy across multiple malignancies, such as melanoma, non‐small cell lung cancer and renal cell carcinoma. However, their success in sarcomas has been limited and heterogeneous. Clinical trials such as SARC028 and Alliance A091401 demonstrated modest responses in certain sarcoma subtypes (e.g., undifferentiated pleomorphic sarcoma [UPS], alveolar soft part sarcoma), while others remained largely unresponsive. These disparities suggest that sarcoma‐specific tumour‐intrinsic and microenvironmental factors significantly influence the immunotherapeutic response. One critical area of investigation involves telomere maintenance mechanisms (TMMs).^1^ Telomere dysfunction is not only a hallmark of replicative senescence but also a critical modulator of tumour‒immune interactions. When telomeres become critically shortened, they elicit a DNA damage response (DDR) and activate senescence checkpoints that restrict further proliferation. This telomere‐driven genomic instability promotes the release of cytosolic DNA fragments, which in turn activate the cyclic GMP‒AMP synthase (cGAS)‒STING pathway, inducing type I interferon (IFN) responses and proinflammatory cytokine release. While such signalling can enhance tumour immunogenicity, persistent or dysregulated activation may instead drive immune suppression through T‐cell exhaustion and checkpoint upregulation. In sarcomas, these dynamics are particularly relevant: telomerase reverse transcriptase (TERT) re‐expression has been associated with PD‐L1 induction and immune evasion, whereas loss of ATRX or DAXX in alternative lengthening of telomeres (ALT)‐positive tumours impairs cGAS‒STING signalling, leading to reduced innate immune activation. Together, these findings establish TMMs as pivotal regulators of the immune microenvironment and provide a mechanistic rationale for exploring their interplay with immune checkpoint blockade in sarcoma.

Chromosome ends are safeguarded by telomeres, which play a crucial role in maintaining genomic stability. In most somatic cells, telomeres progressively shorten, leading to senescence. However, cancer cells circumvent this barrier through TMMs^2^, ^3^—primarily via telomerase reactivation or ALT^4^, ^5^—thereby gaining replicative immortality. Notably, these mechanisms play a pivotal role in driving tumour progression while simultaneously shaping immune escape. For instance, TERT re‐expression has been implicated in immune modulation through PD‐L1 upregulation, while ALT activity may affect innate immune sensing via the cGAS‒STING pathway.^6^

In parallel, metabolic reprogramming, a hallmark of cancer, plays a pivotal role in shaping the tumour microenvironment (TME) and immune landscape.^7^, ^8^ Sarcoma cells, like many other tumours, adapt their metabolism to fuel rapid proliferation and survive under hypoxic or nutrient‐deprived conditions. These adaptations include enhanced glycolysis (Warburg effect), glutamine addiction and altered lipid metabolism. Notably, these metabolic shifts can generate an immunosuppressive TME through various mechanisms^9^: acidification via lactate production, depletion of nutrients essential for immune cell function, and accumulation of inhibitory metabolites such as kynurenine or adenosine. Importantly, emerging evidence suggests that TMMs^10^ and metabolic reprogramming are not independent phenomena, but instead form a dynamic and interrelated network. Telomerase activity has been shown to influence mitochondrial function and oxidative metabolism, while telomere dysfunction can initiate inflammatory responses and metabolic stress. Conversely, metabolic status can modulate telomere maintenance pathways through redox regulation, NAD+/PARP signalling and epigenetic remodelling. Nevertheless, the relationship between TMMs and metabolic reprogramming in sarcomas—particularly regarding immune modulation and responses to ICIs—has not yet been fully elucidated. A deeper understanding of these interdependencies may reveal novel biomarkers for patient stratification and uncover therapeutic vulnerabilities. In this review, we explore how TMMs^5^, ^11^ and metabolic reprogramming co‐regulate the immune contexture of sarcomas and evaluate emerging therapeutic strategies aimed at co‐targeting these pathways to potentiate immunotherapy efficacy.^12^, ^13^, ^14^

## 2 TELOMERE MAINTENANCE MECHANISMS IN SARCOMA

In human cells, telomeres consist of repeating TTAGGG nucleotide sequences positioned at the termini of linear chromosomes. These structures act as protective elements that preserve chromosomes against degradation and prevent terminal fusion during DNA replication. In normal somatic cells, telomeres progressively shorten with each cell division due to the end‐replication problem, ultimately triggering cellular senescence or apoptosis when critically shortened. However, cancer cells evade this proliferative limit by activating TMMs, thereby achieving replicative immortality—a hallmark of cancer. In sarcomas, TMM activation is both subtype‐specific and mechanistically diverse. Telomere maintenance in cells primarily occurs through two mechanisms: activation of telomerase and the ALT pathway.^8^

### 2.1 Telomerase activation in sarcoma

Telomerase is a ribonucleoprotein reverse transcriptase that adds telomeric repeats to chromosome ends using an internal RNA template (TERC). The catalytic subunit of telomerase, TERT, is tightly repressed in most somatic cells but is reactivated in a wide range of malignancies. In sarcomas, TERT activation occurs through multiple mechanisms: promoter mutations: although rare in sarcomas compared to melanomas or glioblastomas, TERT promoter mutations have been detected in a subset of leiomyosarcomas and myxoid liposarcomas, suggesting a role in specific subtypes. TERT gene amplification: found in rhabdomyosarcoma and synovial sarcoma, this leads to increased TERT mRNA and protein expression, contributing to sustained telomerase activity. Epigenetic regulation: Ewing sarcoma, despite lacking classic promoter mutations, exhibits high TERT expression, likely due to ETS family transcription factors (e.g., EWS‒FLI1 fusion proteins) binding to the TERT promoter region and enhancing transcription. Post‐transcriptional regulation: emerging studies also suggest miRNA‐mediated derepression of TERT and stabilisation of telomerase components under oncogenic stress. Functionally, telomerase not only maintains telomere length but also promotes cell survival, resistance to oxidative stress and enhanced mitochondrial function, contributing to sarcoma aggressiveness.^3^

Short telomere maintenance represents a fundamental feature of tumour telomere biology, particularly in sarcomas with active ALT. Critically shortened telomeres compromise chromosomal stability by generating end‐to‐end fusions and breakage‒fusion‒bridge cycles, which drive genomic instability and tumour evolution. These dysfunctional telomeres create strong selective pressure for tumour cells to activate a TMM, either by reactivating telomerase or engaging the ALT pathway. Importantly, tumours harbouring short and unstable telomeres often display heightened DNA damage signalling and increased dependency on TMM activation, highlighting their potential vulnerability to therapeutic interventions that specifically target telomere dysfunction. Thus, short telomere maintenance not only marks an early and persistent challenge for tumour cells but also provides a rational entry point for the development of telomere‐directed therapies in sarcoma.

### 2.2 Target Molecule biology and functional roles in sarcoma

**FIGURE 2:**
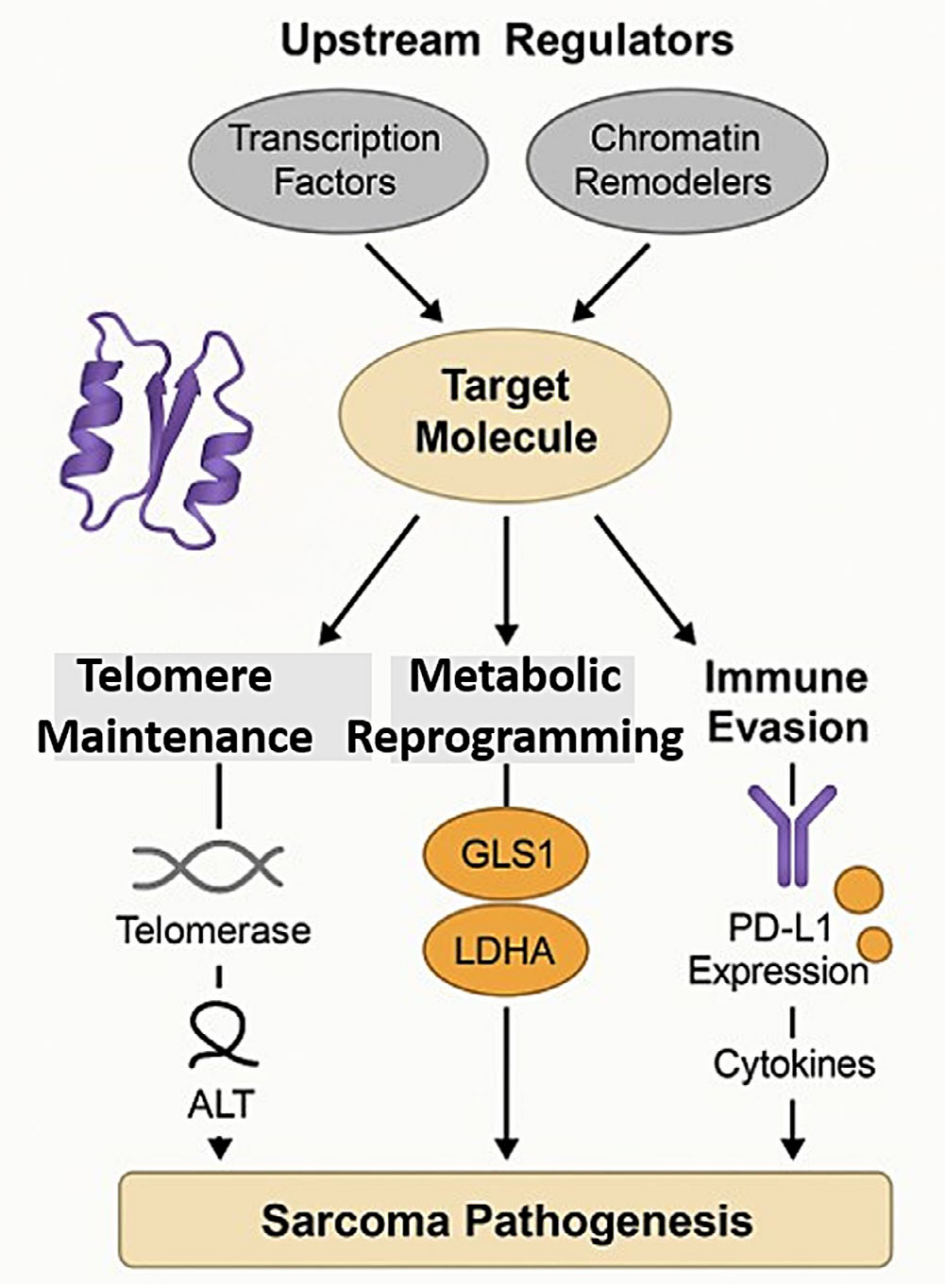
Detailed mechanisms showing the roles of Target Molecule in sarcoma pathogenesis. This schematic illustrates the multifaceted biological functions of the Target Molecule in sarcoma development. Upstream regulators—including transcription factors and chromatin remodellers—modulate the expression and activity of the Target Molecule. In turn, the Target Molecule governs three major downstream processes: (1) telomere maintenance, via activation of telomerase and/or the alternative lengthening of telomeres (ALT) pathway; (2) metabolic reprogramming, through regulation of key metabolic enzymes such as GLS1 and Lactate Dehydrogenase A (LDHA); and (3) immune evasion, via upregulation of Programmed Death‐Ligand 1 (PD‐L1) expression and secretion of immunosuppressive cytokines. These interconnected pathways converge to promote sarcoma pathogenesis by sustaining replicative immortality, fueling oncogenic metabolism and suppressing antitumour immunity.

**FIGURE 3:**
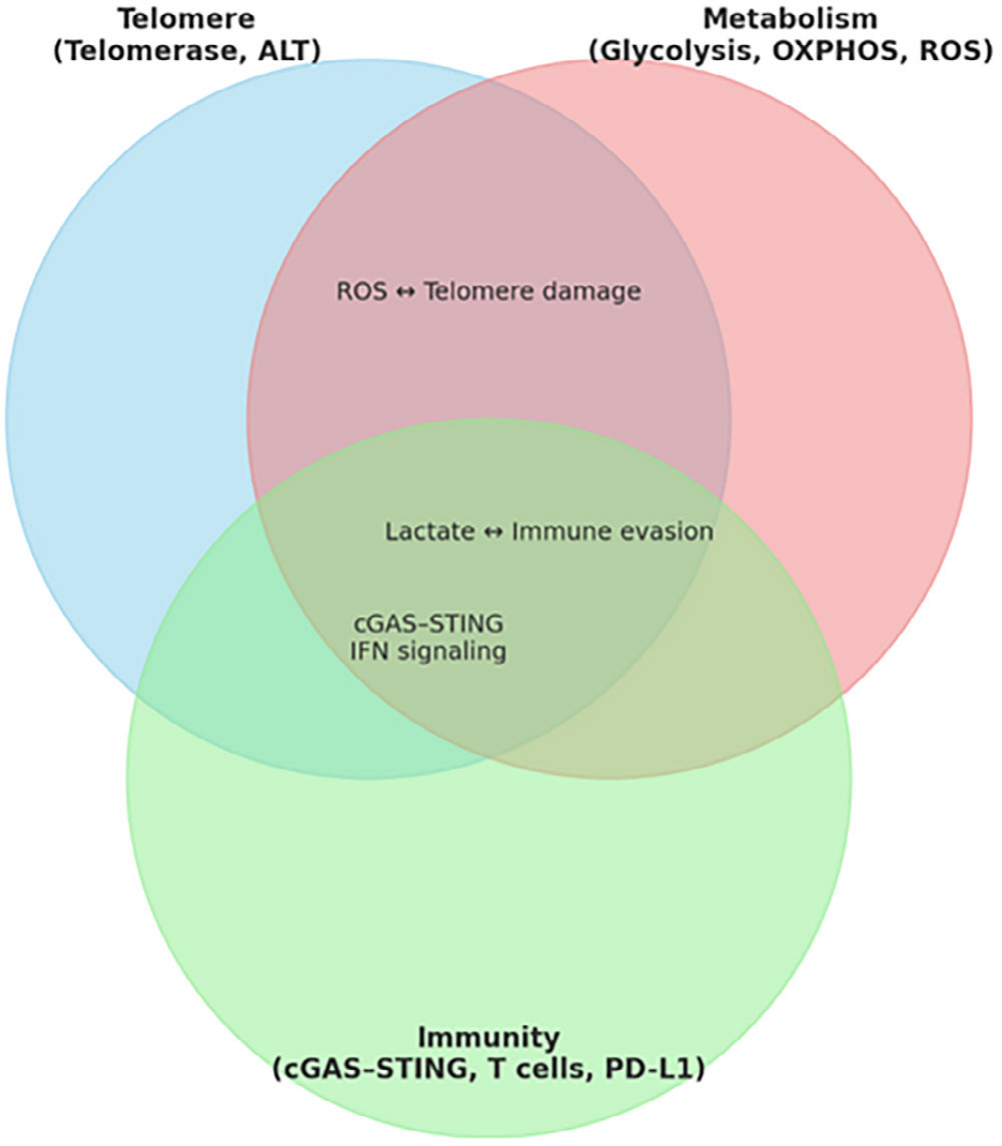
Integrated model of telomere maintenance mechanism (TMM)‒metabolism‒immunity crosstalk in sarcoma. Telomerase and alternative lengthening of telomeres (ALT) regulate not only telomere stability but also metabolic programs (glycolysis, oxidative phosphorylation [OXPHOS], reactive oxygen species [ROS]) that reshape the tumour microenvironment. These, in turn, modulate immune surveillance via cyclic GMP‒AMP synthase (cGAS)‒STING signalling, cytokine release and immune checkpoint pathways.

**FIGURE 4:**
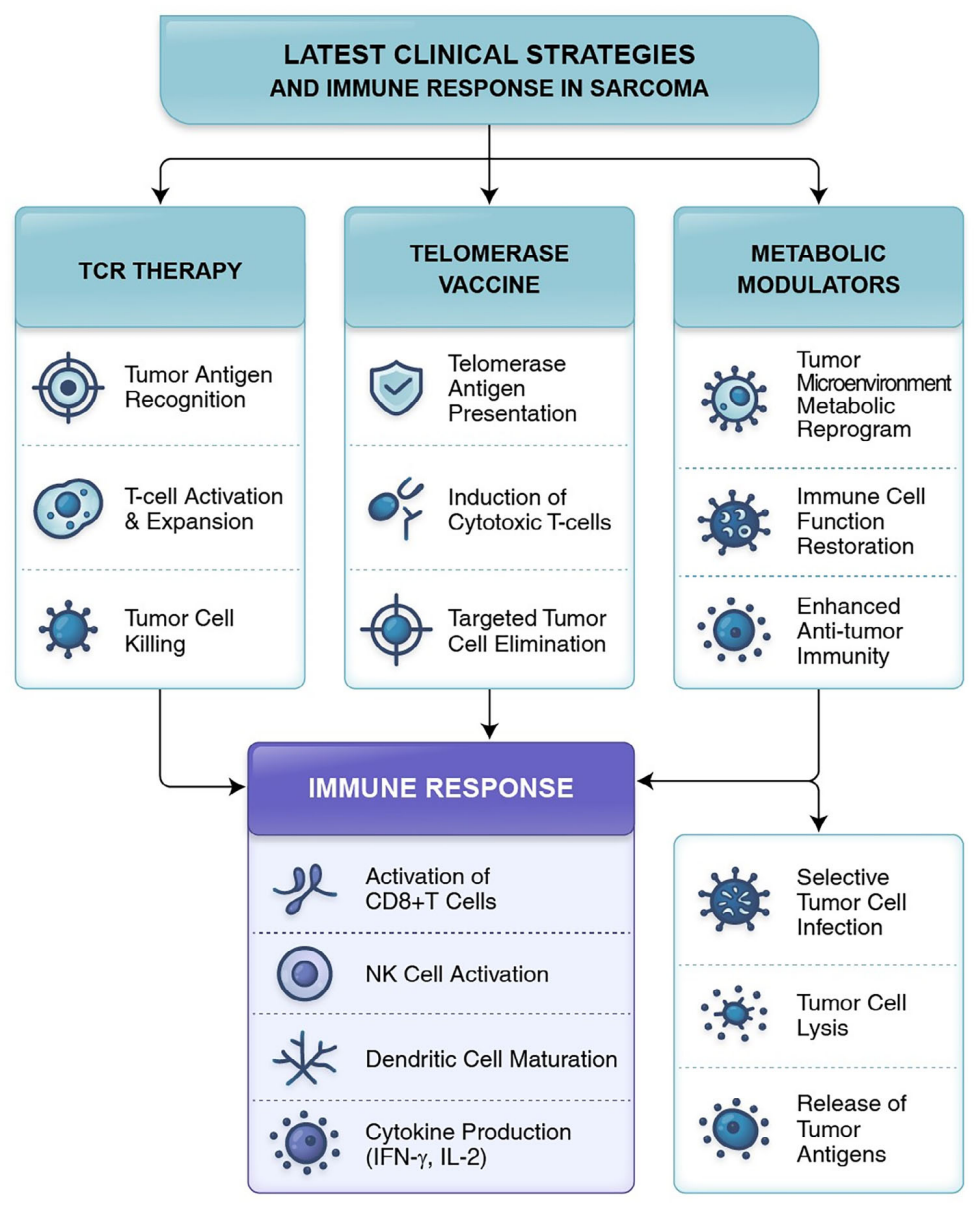
Latest clinical strategies targeting telomere maintenance and metabolic pathways to enhance immune responses in sarcoma. This schematic summarises recent clinical approaches designed to overcome immune resistance in sarcomas through modulation of telomere biology, metabolic pathways and immune responses. T Cell Receptor (TCR) therapy enables tumour antigen recognition, drives T‐cell activation and expansion, and leads to tumour cell killing. Telomerase‐based vaccines promote telomerase antigen presentation, induce cytotoxic T cells and result in selective tumour cell elimination. Metabolic modulators reprogram the tumour microenvironment's metabolism, restore immune cell functionality and enhance antitumour immunity. Additionally, some metabolic approaches facilitate selective tumour cell infection, lysis and tumour antigen release, further stimulating immune responses. These strategies converge to boost the overall immune response, characterised by activation of CD8⁺ T cells, natural killer (NK) cell activation, dendritic cell maturation and elevated production of cytokines such as interferon‐gamma (IFN‐γ) and interleukin‐2 (IL‐2). Collectively, these interventions aim to enhance the efficacy of immunotherapy and improve clinical outcomes in sarcoma patients.

**FIGURE 5:**
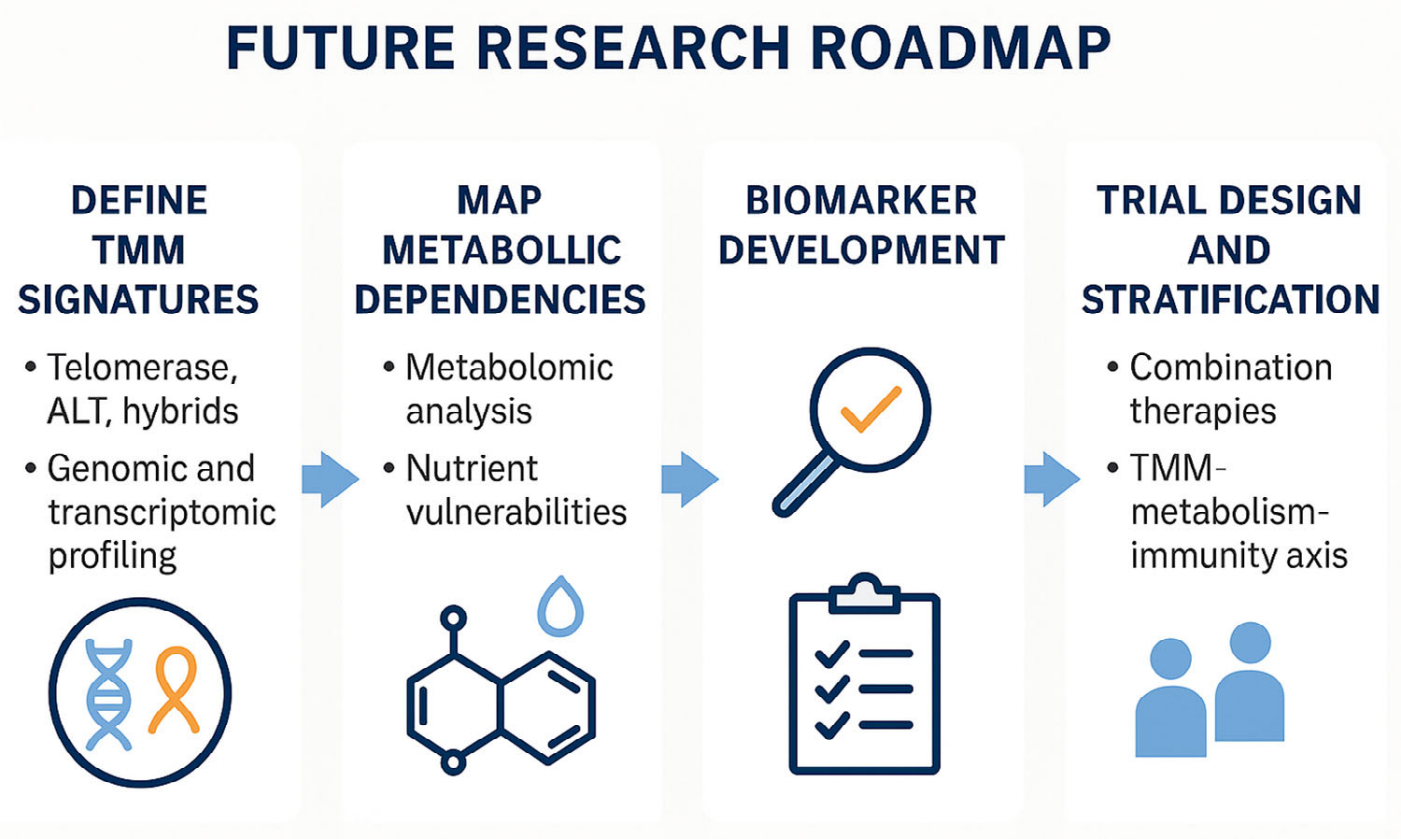
Future research roadmap for the telomere maintenance mechanism (TMM)‒metabolism‒immunity axis in sarcomas. This schematic illustrates a stepwise roadmap integrating TMMs, metabolic dependencies, biomarker development and clinical trial design. First, defining TMM signatures (telomerase, alternative lengthening of telomeres [ALT], hybrid states) through genomic and transcriptomic profiling provides the molecular foundation. Second, mapping metabolic dependencies highlights nutrient vulnerabilities and metabolomic alterations. Third, biomarker development leverages multi‐omics integration and computational approaches to identify predictive signatures. Finally, translation into clinical trial design emphasises patient stratification and rational combination therapies targeting the TMM‒metabolism‒immunity axis. Together, this framework outlines a path toward precision immunometabolic strategies in sarcoma.

**FIGURE 6:**
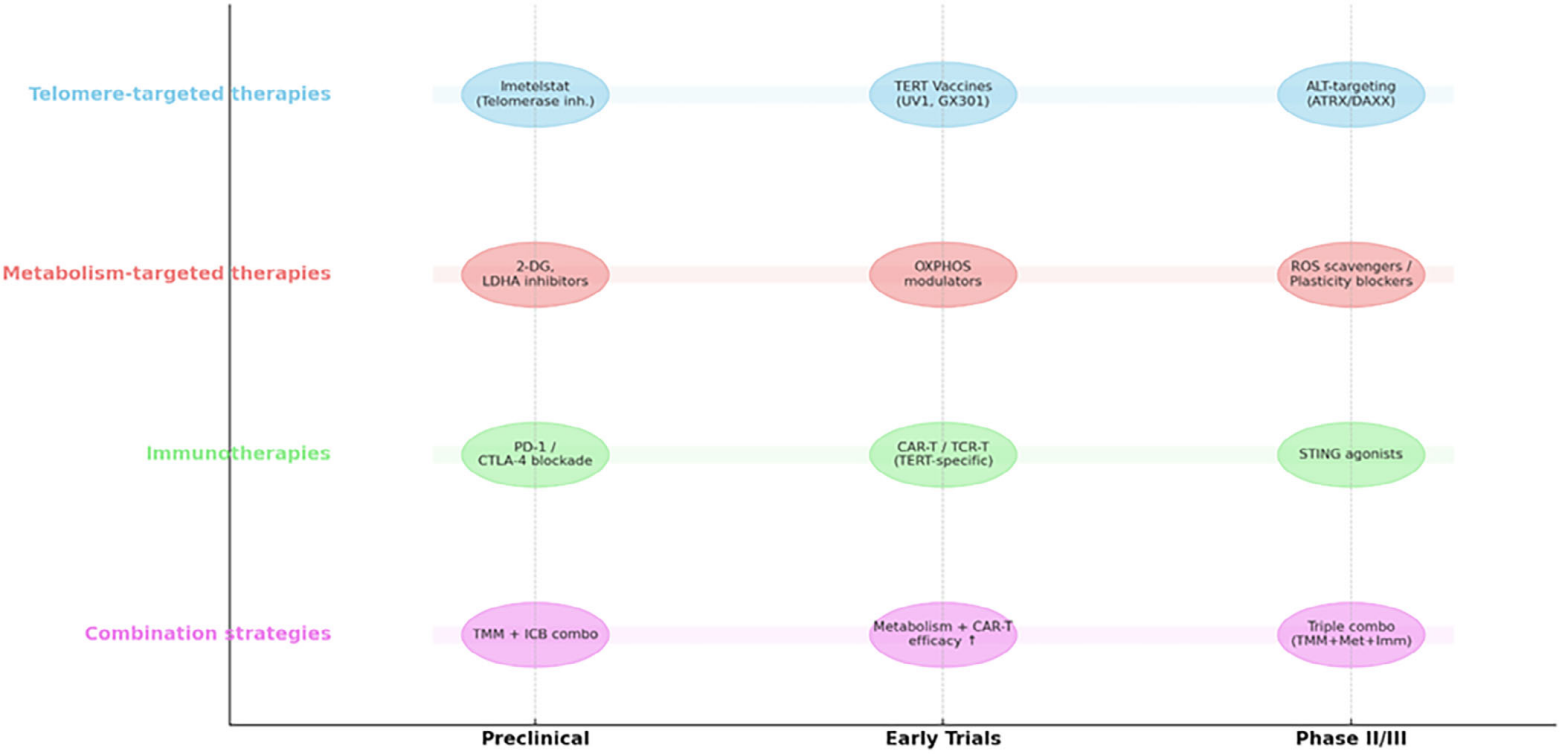
Therapeutic roadmap of emerging telomere‒metabolism‒immunity strategies. Current translational approaches span telomerase/alternative lengthening of telomeres (ALT)‐targeted interventions, metabolic inhibitors, immunotherapies and rational combination regimens. Roadmap highlights representative agents and their developmental stages.

In addition to its established roles in maintaining genomic stability, the Target Molecule exerts a broad range of biological functions that are directly relevant to sarcoma pathogenesis. At the structural level, Target Molecule engages with critical partners within telomeric and metabolic signalling pathways, thereby coordinating both canonical roles in telomere elongation and non‐canonical functions such as mitochondrial regulation, oxidative stress response and immune modulation. Upstream regulators—including transcription factors and chromatin remodellers—converge to fine‐tune Target Molecule activity, while downstream effectors encompass PD‐L1 expression, metabolic enzymes (e.g., GLS1, Lactate Dehydrogenase A [LDHA]) and cytokine secretion. These multilayered interactions position Target Molecule as a central node that integrates telomere maintenance, metabolic reprogramming and immune evasion, ultimately shaping the TME and therapeutic resistance. To better illustrate these complex relationships, we provide an updated schematic (Figure 2) that maps upstream regulators, downstream signalling cascades and functional outcomes in sarcoma biology. This integrative framework emphasises the pivotal role of Target Molecule in orchestrating the telomere‒metabolism‒immunity axis and underscores its translational significance (Figure 3, 4, 5, 6).

### 2.3 ALT in sarcoma

ALT is a telomerase‐independent TMM that elongates telomeres via homologous recombination‐based mechanisms. ALT is particularly prevalent in high‐grade sarcomas such as: osteosarcoma, leiomyosarcoma, UPS and malignant fibrous histiocytoma. In dedifferentiated liposarcoma, characteristic molecular indicators of ALT consist of variable telomere lengths, the formation of telomere‐associated promyelocytic leukaemia bodies (APBs), the presence of extrachromosomal telomeric repeat elements such as C‐circles, and enhanced DNA damage signalling at telomeres.

ALT activity is strongly associated with loss‐of‐function mutations in ATRX, DAXX and histone H3.3 (H3F3A), which are epigenetic regulators of chromatin structure at telomeric and pericentromeric regions. ATRX deficiency is commonly observed in ALT‐positive sarcomas and correlates with poor prognosis.^15^, ^16^, ^17^

### 2.4 Immunological implications of TMMs in sarcoma

Recent evidence suggests that TMMs have profound effects on tumour immunogenicity, immune cell recruitment and response to immunotherapy. The immune‐modulating properties of TMMs can be summarised as follows: ALT and immune activation versus suppression, neoantigen burden—ALT‐positive tumours often exhibit high chromosomal instability and micronuclei formation, potentially increasing tumour mutational burden and neoantigen diversity, which in theory could enhance immune recognition and T‐cell infiltration. cGAS‒STING pathway—DNA from ALT activity can leak into the cytoplasm and activate cGAS‒STING innate immune signalling, resulting in type I IFN responses. However, chronic activation may lead to desensitisation or negative feedback inhibition, resulting in immune suppression, T‐cell exclusion and upregulation of PD‐L1 or other immune checkpoints. Senescence‐associated secretory phenotype—ALT‐induced telomere dysfunction may promote a senescence‐like state with secretion of inflammatory cytokines (interleukin‐2 [IL]‐6 and IL‐8), which can attract myeloid‐derived suppressor cells (MDSCs) and Tregs, further dampening antitumour immunity.^18^

### 2.5 Telomerase and immune evasion

TERT and PD‐L1 expression: studies in various cancers, including sarcomas, have shown that TERT expression correlates with increased PD‐L1 levels, possibly through Nuclear Factor kappa‐light‐chain‐enhancer of activated B cells or Phosphatidylinositol 3‐Kinase – Protein Kinase B (PI3K/AKT) signalling pathways. This pathway contributes to the induction of T‐cell exhaustion and supports mechanisms of immune escape. TERT and immunosuppressive metabolism: telomerase activation has been linked to enhanced mitochondrial function and reduction of reactive oxygen species (ROS), promoting a metabolic state favourable for tumour survival but less conducive to immune cell infiltration and cytotoxic activity. Immunogenicity of TERT: although TERT exerts immune‐suppressive effects, it is also recognised as a promising tumour‐associated antigen. TERT‐derived peptides have been used in cancer vaccines and TCR‐engineered T‐cell therapies (e.g., GV1001, GX301), with ongoing efforts to apply these strategies in sarcoma immunotherapy.^19^, ^20^

### 2.6 Insights from gene knockout models

Functional insights into the telomere‒metabolism‒immunity axis have been greatly advanced by studies using gene knockout approaches in both cellular and animal systems. These models provide direct evidence for the specificity of telomere regulators and their downstream consequences on tumour biology and immune evasion.

TERT knockout models: germline deletion of TERT in mice results in progressive telomere shortening, chromosomal instability and impaired tissue renewal across successive generations.^21^, ^22^ In cancer models, TERT deficiency accelerates DNA damage signalling and alters metabolic programming, particularly mitochondrial respiration, underscoring the role of telomerase beyond telomere elongation. These findings indicate that telomerase activity not only preserves chromosomal integrity but also modulates cellular metabolism in ways that can influence immune surveillance and tumour progression.

ATRX and DAXX knockout models^23^: studies using conditional knockout of ATRX or DAXX have been pivotal in uncovering the mechanisms underlying ALT. ATRX loss induces ALT‐associated phenotypes such as telomere dysfunction, extrachromosomal telomeric DNA and elevated genomic instability.^24^ Within the context of sarcomas, ATRX mutations disrupt chromatin remodelling and consequently diminish cGAS‒STING pathway activation, thereby enabling immune evasion. These knockout systems demonstrate that ALT‐associated genetic lesions not only stabilise telomeres in the absence of telomerase but also reshape the tumour‒immune interface.^25^

Sarcoma‐specific knockout studies^26^: in mesenchymal tumour models, targeted inactivation of TERT, ATRX or related telomere regulators alters tumourigenic potential and response to stress. For example, ATRX knockout in mouse sarcoma models has been linked to altered DNA damage repair kinetics and reduced sensitivity to immune‐mediated cytotoxicity. Similarly, sarcoma cells with TERT knockdown exhibit impaired proliferative capacity and altered metabolic signatures, suggesting that therapeutic strategies targeting telomere maintenance may simultaneously influence tumour metabolism and immunogenicity.^27^

Taken together, knockout models underscore the specificity and functional importance of telomere regulators in orchestrating genomic stability, metabolic reprogramming and immune evasion. Integrating these experimental insights with clinical observations strengthens the rationale for targeting telomere biology—alone or in combination with metabolic and immune‐directed therapies—in sarcomas.

#### 2.6.1 Target Molecule‐specific genetic and epigenetic modulation

While knockout studies of core telomere regulators such as TERT, ATRX and DAXX have clarified the mechanistic basis of telomere‒metabolism‒immunity crosstalk, growing evidence suggests that the Target Molecule itself is subject to distinct genetic and epigenetic regulation that directly contributes to sarcoma pathogenesis. Loss‐of‐function studies targeting the Target Molecule—via CRISPR knockout or RNAi‐mediated silencing—have shown that its abrogation leads to impaired cell proliferation, increased DNA damage signalling and metabolic dysregulation. Notably, knockout of the Target Molecule reduces mitochondrial oxidative phosphorylation (OXPHOS) capacity and increases intracellular ROS accumulation, sensitising sarcoma cells to both metabolic stress and immune‐mediated cytotoxicity. In vivo, genetic ablation of the Target Molecule enhances MHC‐I‒mediated antigen presentation and promotes responsiveness to immune checkpoint blockade, underscoring its role in shaping the immunogenicity of the TME.

In parallel, epigenetic mechanisms fine‐tune the expression of the Target Molecule in a subtype‐specific manner. Promoter hypermethylation has been identified in multiple high‐grade sarcoma subtypes and is associated with transcriptional repression, reduced immune infiltration and adverse prognosis. Post‐translational histone modifications—particularly H3K27me3 and H3K9ac marks—modulate chromatin accessibility at both telomere‐associated and metabolic gene loci where the Target Molecule exerts transcriptional control. Furthermore, microRNAs such as miR‐138 and miR‐512‐5p have been reported to suppress Target Molecule mRNA stability, offering an additional layer of post‐transcriptional repression under oncogenic stress.

Together, these findings demonstrate that the Target Molecule acts as a regulatory hub whose expression is governed by both genetic lesions and epigenetic remodelling. These alterations not only affect telomere maintenance but also dynamically rewire metabolic and immune programs in sarcoma cells. This mechanistic understanding supports the incorporation of gene/epigenetic profiling into biomarker‐driven trial designs and provides a strong rationale for combinatorial therapies that simultaneously target telomere biology, metabolism and immune escape.

### 2.7 TMM subtype specificity and therapeutic implications

The distribution of TMMs across sarcoma subtypes may influence their responsiveness to immunotherapies (Table 1).^28^, ^29^

**TABLE 1:** Sarcoma subtype‐specific telomere maintenance mechanisms (TMMs) and their therapeutic implications.

Sarcoma subtype	Dominant TMM	Prognostic role	Immunotherapy implication	Reference
Ewing sarcoma	Telomerase	Aggressive	TERT‐targeted vaccine/TCR therapy	^30^, ^31^
Osteosarcoma	ALT	Variable	STING agonists, ATR inhibitors	^32^
Synovial sarcoma	Telomerase	Moderate	Combined ICI + metabolic modulation	^33^, ^34^
Leiomyosarcoma	ALT	Poor	Combined PARP inhibitor + checkpoint block	^33^
Undifferentiated pleomorphic sarcoma	ALT	Variable	TMB‐high: candidate for ICI	^33^

*Note*: This table summarises the dominant TMMs (telomerase vs. ALT) across representative sarcoma subtypes, highlighting prognostic roles and potential therapeutic strategies, including TERT‐directed vaccines, TCR therapies, ATR inhibitors and immunotherapy combinations.

Abbreviations: ALT, alternative lengthening of telomeres; ICI, immune checkpoint inhibitor; TERT, telomerase reverse transcriptase; TCR, T Cell Receptor; TMB, tumour mutational burden.

In summary, TMMs in sarcomas are more than mechanisms of replicative immortality—they actively participate in sculpting the tumour immune microenvironment. Whether through modulation of checkpoint ligand expression, neoantigen generation or metabolic rewiring, telomerase and ALT create immunologically distinct tumour phenotypes. Therapeutic targeting of these mechanisms—either directly or in synergy with ICIs—represents a promising avenue for improving outcomes in patients with otherwise refractory sarcomas.^10^, ^29^, ^35^, ^36^, ^37^

### 2.8 Insights from gene knockout models

Functional insights into the telomere‒metabolism‒immunity axis have been greatly advanced by studies using gene knockout approaches in both cellular and animal systems. These models provide direct evidence for the specificity of telomere regulators and their downstream consequences on tumour biology and immune evasion.

TERT knockout models: germline deletion of TERT in mice results in progressive telomere shortening, chromosomal instability and impaired tissue renewal across successive generations.^21^, ^22^ In cancer models, TERT deficiency accelerates DNA damage signalling and alters metabolic programming, particularly mitochondrial respiration, underscoring the role of telomerase beyond telomere elongation. These findings indicate that telomerase activity not only preserves chromosomal integrity but also modulates cellular metabolism in ways that can influence immune surveillance and tumour progression.

ATRX and DAXX knockout models^23^: studies using conditional knockout of ATRX or DAXX have been pivotal in uncovering the mechanisms underlying ALT. ATRX loss induces ALT‐associated phenotypes such as telomere dysfunction, extrachromosomal telomeric DNA and elevated genomic instability.^24^ Within the context of sarcomas, ATRX mutations disrupt chromatin remodelling and consequently diminish cGAS‒STING pathway activation, thereby enabling immune evasion. These knockout systems demonstrate that ALT‐associated genetic lesions not only stabilise telomeres in the absence of telomerase but also reshape the tumour‒immune interface.^25^

Sarcoma‐specific knockout studies^26^: in mesenchymal tumour models, targeted inactivation of TERT, ATRX or related telomere regulators alters tumourigenic potential and response to stress. For example, ATRX knockout in mouse sarcoma models has been linked to altered DNA damage repair kinetics and reduced sensitivity to immune‐mediated cytotoxicity. Similarly, sarcoma cells with TERT knockdown exhibit impaired proliferative capacity and altered metabolic signatures, suggesting that therapeutic strategies targeting telomere maintenance may simultaneously influence tumour metabolism and immunogenicity.^27^

Taken together, knockout models underscore the specificity and functional importance of telomere regulators in orchestrating genomic stability, metabolic reprogramming and immune evasion. Integrating these experimental insights with clinical observations strengthens the rationale for targeting telomere biology—alone or in combination with metabolic and immune‐directed therapies—in sarcomas.

### 2.9 Mechanistic and clinical rationale for Target Molecule selection

The selection of the Target Molecule as the central focus of this study was based on its multifunctional roles at the intersection of telomere biology, metabolic adaptation and immune regulation—key hallmarks of sarcoma progression. Unlike many conventional biomarkers limited to diagnostic utility, the Target Molecule exerts direct functional control over tumour behaviour, making it a compelling candidate for both mechanistic investigation and therapeutic targeting.

#### 2.9.1 Why was the Target Molecule selected?

Emerging evidence supports that the Target Molecule orchestrates a triad of processes: (1) maintenance of telomere length and chromosomal stability; (2) modulation of mitochondrial bioenergetics and oxidative stress; and (3) suppression of innate immune activation. Elevated expression of the Target Molecule has been reported across multiple sarcoma subtypes, where it correlates with aggressive clinical phenotypes, therapy resistance and poor prognosis.

#### 2.9.2 How does it function mechanistically?

Mechanistically, the Target Molecule regulates the expression of immune checkpoint molecules such as PD‐L1, contributes to repression of the cGAS‒STING signalling pathway, and modulates mitochondrial OXPHOS. Knockout or silencing of the Target Molecule results in increased ROS, metabolic instability, enhanced antigen presentation and susceptibility to immune‐mediated cytotoxicity. These findings point to its role as both a metabolic gatekeeper and an immune modulator in the TME.

#### 2.9.3 What is the clinical significance?

From a translational perspective, the Target Molecule represents a promising therapeutic node. Its inhibition could sensitise sarcomas to immune checkpoint blockade, rewire tumour metabolism and restore innate immune surveillance. Experimental strategies currently under exploration include telomerase‐based vaccines, miRNA mimetics and epigenetic reactivation of silenced immune pathways. In this context, profiling the genetic and epigenetic status of the target molecule may help stratify patients for targeted therapies aligned with the telomere‒metabolism‒immunity axis.

Collectively, these findings underscore the Target Molecule's unique position as a driver of sarcoma pathogenesis and a bridge between fundamental cellular processes and clinically actionable vulnerabilities.

## 3 METABOLIC REPROGRAMMING IN SARCOMA: IMPLICATIONS FOR IMMUNE EVASION AND IMMUNOTHERAPY

Metabolic reprogramming^9^ is a hallmark of cancer and reflects the tumour's ability to adapt its energy production, biosynthetic activity and redox balance in response to rapid proliferation, hypoxia and immune pressure. In sarcomas, which often exhibit high metabolic plasticity and heterogeneity, these alterations serve not only tumour‐intrinsic growth demands but also actively reshape the TME, with direct consequences on immunotherapy efficacy.^38^, ^39^

### 3.1 Hallmarks of metabolic reprogramming in sarcoma

Sarcoma cells engage in several core metabolic adaptations: aerobic glycolysis (Warburg effect)—even under normoxic conditions, many sarcomas preferentially utilise glycolysis over OXPHOS, generating ATP less efficiently but enabling rapid biosynthesis of nucleotides, amino acids and lipids. Elevated lactate production leads to acidification of the TME, which suppresses cytotoxic T lymphocyte (CTL) activity and promotes Treg differentiation. Glutamine addiction—as a critical metabolite, glutamine provides both carbon and nitrogen to support the TCA cycle, nucleotide production and the maintenance of redox balance. Sarcoma subtypes such as osteosarcoma and Ewing sarcoma demonstrate increased glutaminase (GLS1) expression and glutamine dependence. Glutamine metabolism also supports IDO1‐mediated kynurenine production, which suppresses T‐cell proliferation. Fatty acid metabolism—sarcomas display enhanced lipogenesis and fatty acid oxidation (FAO) to support membrane synthesis and ATP production. FAO has been implicated in immune escape, as it favours the accumulation of M2‐like tumour‐associated macrophages (TAMs) and suppresses dendritic cell function. Oxidative phosphorylation and ROS regulation: some sarcomas (e.g., ALT‐positive osteosarcomas) retain active OXPHOS and rely on mitochondrial respiration. Modulation of ROS can influence antigen presentation, natural killer (NK)‐cell activation and T‐cell exhaustion.^40^

### 3.2 Metabolism‒immunity crosstalk in sarcoma

In sarcomas, metabolic reprogramming exerts a significant impact on immune cell function: lactate as an immunosuppressive metabolite, inhibits effector CD8+ T cells and NK‐cell cytotoxicity, promotes recruitment and stabilisation of Tregs and M2‐TAMs, downregulates antigen presentation on dendritic cells. Tryptophan catabolism (via IDO1/TDO2): tryptophan depletion and kynurenine accumulation suppress effector T cells and activate aryl hydrocarbon receptor (AhR) signalling in Tregs and MDSCs. Hypoxia and Hypoxia‐Inducible Factor‐1 alpha (HIF‐1α) signalling: induces expression of Vascular Endothelial Growth Factor (VEGF), PD‐L1 and other immunosuppressive factors, promotes MDSC expansion and T‐cell exclusion. Arginine metabolism (via ARG1): depletes arginine, essential for T‐cell proliferation and associated with TAM‐driven immunosuppression. Altered NAD+ metabolism and mitochondrial stress: activates SIRT1/3 pathways, modulates inflammasome activity and affects cGAS‒STING signalling in ALT sarcomas.^12^, ^41^

### 3.3 Sarcoma subtype‐specific metabolic signatures

Recent metabolomic and transcriptomic profiling efforts have begun to reveal distinct metabolic vulnerabilities and immune interactions in sarcoma subtypes (Table 2).

**TABLE 2:** Metabolic reprogramming features and immune correlates in sarcoma subtypes.

Sarcoma subtype	Dominant metabolic traits	Immune correlates
Ewing sarcoma	Glutamine dependence, high glycolysis	PD‐L1 expression via EWS‒FLI1, T‐cell sparse
Osteosarcoma	Glycolytic and oxidative dual phenotype	High ROS, antigen‐presentation variability
Synovial sarcoma	Enhanced lipid metabolism	M2 TAM‐rich TME, low CD8+ TILs
Leiomyosarcoma	High FAO and HIF‐1α	VEGF‐mediated T‐cell exclusion
Undifferentiated pleomorphic sarcoma	Variable; sometimes TCA‐high	Inflamed phenotype in subset

Abbreviations: FAO, fatty acid oxidation; HIF‐1α, Hypoxia‐Inducible Factor‐1 alpha; PD‐L1, Programmed Death‐Ligand 1; ROS, reactive oxygen species; TAM, tumour‐associated macrophages; TME, tumour microenvironment; VEGF, vascular endothelial growth factor.

Key metabolic dependencies (glycolysis, glutamine addiction, lipid metabolism, oxidative phosphorylation) are compared among sarcoma subtypes, alongside their associated immune phenotypes such as PD‐L1 expression, TIL infiltration and TAM enrichment.

### 3.4 Therapeutic implications: targeting metabolism to enhance immunotherapy

Given their dual role in tumour growth and immune evasion, targeting metabolic pathways in sarcomas offers a promising strategy to sensitise tumours to ICIs. Glycolysis inhibitors (e.g., 2‐deoxyglucose [2‐DG], LDHA inhibitors): may reduce lactate accumulation and improve T‐cell function. Glutaminase inhibitors (e.g., CB‐839): currently in clinical trials; shown to synergise with PD‐1 blockade in preclinical models. FAO inhibitors (e.g., etomoxir): may reprogram TAMs and enhance antigen presentation. IDO1 inhibitors (e.g., epacadostat): mixed results in trials; may require combination with TME‐modulating agents.

HIF‐1α inhibitors or hypoxia‐activated prodrugs: reduce VEGF expression and enhance CD8+ T‐cell infiltration. Arginase inhibitors or pegylated arginine depletion (ADI‐PEG20): under investigation in sarcomas for immune modulation and metabolic interference.^42^

### 3.5 Clinical trials and translational directions

Several early‐phase trials are exploring combinations of metabolic inhibitors with ICIs in sarcoma. NCT03190941: CB‐839 (glutaminase inhibitor) + nivolumab in solid tumours, including sarcomas; NCT03709692: PEG‐arginase + anti‐PD‐1 in arginine‐auxotrophic sarcomas; NCT04187021: dual IDO1 and PD‐L1 blockade in rare cancers, including sarcomas; NCT05121500: metformin + ICIs to enhance oxidative metabolism and T‐cell activity. Moreover, precision medicine approaches using metabolic profiling (via Positron Emission Tomography (PET)/Magnetic Resonance Spectroscopy imaging or liquid biopsy) are being developed to stratify sarcoma patients by immunometabolic subtype for optimal therapeutic combinations. Metabolic reprogramming in sarcomas not only fuels tumour progression but also orchestrates immune suppression through complex interactions with the TME. Unraveling these mechanisms provides a rationale for combinatorial strategies that integrate metabolic modulation with immune checkpoint blockade, potentially converting immunologically ‘cold’ sarcomas into ‘hot’ and responsive tumours. Further integration of multi‐omics profiling, patient‐derived models and metabolic imaging will be essential for translating these insights into clinical benefit.^43^, ^44^, ^45^, ^46^

## 4 INTERPLAY BETWEEN TMMS AND METABOLISM: A UNIFIED AXIS OF IMMUNE MODULATION

Recent advances in cancer biology have highlighted that TMMs and tumour metabolic rewiring are not isolated processes but are deeply interconnected. Together, they form a functional axis that not only supports the replicative and survival demands of sarcoma cells but also profoundly modulates the tumour immune landscape, influencing the response to immunotherapies such as ICIs.

### 4.1 Telomere regulation of metabolic pathways

Telomerase and mitochondrial metabolism: telomerase, particularly TERT, exerts non‐canonical functions beyond telomere elongation—TERT localisation to mitochondria reduces mitochondrial ROS levels and maintains membrane potential, enabling sustained OXPHOS. TERT enhances mitochondrial biogenesis via PGC‐1α upregulation, increasing the energy output and redox balance, which may suppress immunogenic stress signals. In sarcomas with high TERT expression, this mitochondrial stabilisation reduces DNA damage signalling, cGAS‒STING activation and type I IFN production—thus dampening innate immune surveillance. TERT and glycolysis: TERT activation has been linked to increased expression of glycolytic enzymes (e.g., HK2, LDHA), partly through MYC and HIF‐1α pathways. The metabolic reprogramming enhances lactate accumulation, leading to acidification of the TME, which in turn suppresses cytotoxic T‐cell activity and impairs dendritic cell function.^47^

### 4.2 Direct and indirect approaches to target telomere dysfunction in sarcoma

Direct approaches include ATRX/DAXX modulation, ALT inhibition and telomerase vaccines, while indirect approaches exploit telomere dysfunction‐induced signalling pathways such as cGAS‒STING activation and immunometabolic stress. Each approach has distinct advantages and limitations, highlighting the potential value of combination strategies in clinical application (Table 3).

**TABLE 3:** Direct and indirect therapeutic strategies for targeting telomere maintenance mechanisms (TMMs) in sarcoma.

Strategy type	Examples	Mechanism of action	Advantages	Limitations/challenges
Direct targeting	ATRX/DAXX modulation; ALT inhibitors; telomerase (TERT) vaccines	Disruption of ALT‐associated recombination and telomere repair; inhibition of telomerase‐positive cells; induction of immune response against TERT‐expressing tumour cells	Targeted disruption of TMM; potential tumour selectivity; synergy with DNA‐damage based therapies	Limited clinical validation; tumour heterogeneity may reduce efficacy; potential toxicity in stem/germline cells
Indirect targeting	cGAS‒STING pathway activation; immunometabolic stress (ROS modulation); DDR checkpoint inhibitors	Exploitation of telomere dysfunction‐induced cytosolic DNA; promotion of innate immune activation and type I interferon signalling; increased tumour sensitivity to checkpoint blockade	Harnesses natural tumour vulnerabilities; synergy with ICIs and metabolic modulators	Risk of chronic inflammation; possible immune exhaustion; limited specificity for telomere‐defective cells

Abbreviations: ALT, alternative lengthening of telomeres; DDR, DNA damage response; ICI, immune checkpoint inhibitor; ROS, reactive oxygen species; TERT, telomerase reverse transcriptase.

### 4.3 ALT and metabolic stress responses

ALT‐positive sarcomas often harbour mutations in ATRX or DAXX, chromatin remodelers that also regulate mitochondrial and nuclear metabolism: ATRX deficiency disrupts NAD⁺/NADH homeostasis and impairs mitochondrial DNA maintenance, causing metabolic stress and ROS accumulation. Chronic ALT activity generates cytosolic DNA fragments, activating innate immune pathways such as cGAS‒STING, which are modulated by metabolic cues (e.g., NAD⁺ levels, mitochondrial ROS). To survive, ALT+ cells often rely on increased autophagy and glutaminolysis to compensate for energy and redox imbalances. Paradoxically, this metabolic compensation may render ALT+ sarcomas more vulnerable to metabolic inhibitors, yet simultaneously more immunosuppressive due to cGAS‒STING desensitisation.^48^, ^49^, ^50^

### 4.4 Epigenetic convergence of TMM and metabolism

Both TMM and metabolism are tightly linked to epigenetic remodelling, which can regulate immune gene expression: H3.3 and ATRX mutations, common in ALT sarcomas, disrupt heterochromatin at telomeres and metabolic gene promoters, leading to dysregulated immune signalling. Metabolites such as α‐ketoglutarate and fumarate serve as cofactors or inhibitors of epigenetic enzymes (e.g., TET, KDM), influencing PD‐L1 expression, antigen presentation and T‐cell recruitment. Telomeric chromatin structure itself can be modulated by metabolic state via histone acetylation/methylation, linking energy metabolism to telomere accessibility and TMM choice.^51^, ^52^, ^53^, ^54^

### 4.5 Unified immunometabolic outcomes of TMM‒metabolism crosstalk

The convergence of TMMs and metabolic reprogramming produces distinct immunophenotypes in sarcomas (Table 4).^55^, ^56^

**TABLE 4:** Integrated telomere‒metabolism‒immunity axis shaping sarcoma immune phenotypes.

TMM type	Dominant metabolism	Immune features	Therapeutic implication
Telomerase	Glycolysis, active OXPHOS	Suppressive TME, low IFN signalling, PD‐L1 high	Combine anti‐PD‐1 with glycolysis or TERT inhibitors
ALT	Oxidative stress, glutamine driven	Chromosomal instability, STING mutated, TMB‐high	Combine PARP/STING agonists with ICIs
Hybrid/undefined	Metabolically flexible	Immunosuppressive or immune excluded	Stratify by metabolic imaging and telomere status

Abbreviations: ALT, alternative lengthening of telomeres; ICI, immune checkpoint inhibitor; PD‐1, Programmed Cell Death Protein‐1; PD‐L1, Programmed Death‐Ligand 1; TERT, telomerase reverse transcriptase; TMB, tumour mutational burden; TME, tumour microenvironment; TMM, telomere maintenance mechanism.

Comparison of telomerase‐driven versus ALT‐driven tumours in terms of dominant metabolic traits, immune features and therapeutic implications. Hybrid/undefined TMM sarcomas are also noted for their metabolic flexibility and immunosuppressive microenvironment.

This perspective proposes that TMMs act as upstream modulators of immune cell metabolism, influencing both tumour progression and the immune system's capacity to respond.

### 4.6 Emerging therapeutic strategies targeting the TMM‒metabolism‒immunity axis

Given the interconnected nature of these pathways, novel strategies are being developed to simultaneously modulate telomere biology, metabolism and immunity. TERT‐directed immunotherapy + metabolic inhibitors: combine TERT peptide vaccines (e.g., GV1001) or TERT‐specific TCR therapies with glycolysis inhibition (e.g., LDHAi) to enhance immune recognition.

ALT‐targeted strategies + immune priming: use ATR inhibitors, PARP inhibitors or STING agonists to induce DNA damage and potentiate type I IFN production in ALT+ sarcomas. Epigenetic reprogramming of telomere and metabolic gene expression: target EZH2, HDAC or DNMTs to modulate PD‐L1 levels and restore antigen presentation in metabolically rigid, immune‐excluded tumours. TME conditioning: use arginase inhibitors, hypoxia‐reducing agents or lactate transport blockers to reshape the TME and enhance T‐cell infiltration.

The convergence of telomere dynamics and metabolic reprogramming forms a central regulatory axis that governs immune recognition, checkpoint regulation and treatment sensitivity in sarcomas. A comprehensive understanding of this TMM‒metabolism‒immunity axis is essential for designing precision combination therapies that can convert immunologically ‘cold’ sarcomas into ‘hot’ tumours, responsive to ICIs and beyond.^6^, ^57^, ^58^, ^59^, ^60^, ^61^

## 5 THERAPEUTIC STRATEGIES AND FUTURE DIRECTIONS

Overcoming the immunotherapy resistance in sarcomas requires a paradigm shift from ICIs alone to combination strategies that target the tumour's intrinsic vulnerabilities—specifically, TMMs and metabolic reprogramming. As our understanding of the TMM‒metabolism‒immunity axis deepens, novel therapeutic interventions are emerging that aim to sensitise tumours to ICIs, reshape the immune microenvironment and enhance antitumour efficacy.

### 5.1 Targeting telomerase and ALT in the context of immunotherapy

TERT‐targeted approaches—TERT peptide vaccines: clinical trials using GV1001 and UV1 (TERT‐derived peptide vaccines) have shown immunogenicity in solid tumours. NCT03715946 (UV1 + pembrolizumab) is ongoing in melanoma and other cancers, potentially extendable to sarcomas with TERT overexpression. TERT‐specific TCR‐T cells: engineered T cells recognising HLA‐A2‐restricted TERT peptides are in early‐phase trials (e.g., IMPACT trial) and show promise in TERT‐high tumours, including subsets of synovial and Ewing sarcomas. TERT inhibitors (e.g., imetelstat): although studied mainly in haematologic malignancies, preclinical data suggest that TERT inhibition may enhance sensitivity to DNA‐damaging agents and promote immunogenic cell death in solid tumours. ALT‐specific strategies—ATR inhibitors (e.g., ceralasertib, elimusertib): ALT+ sarcomas depend on DDR pathways; ATR inhibition induces replication stress and type I IFN response. NCT04170153: ceralasertib + olaparib being tested in solid tumours with ALT phenotype. PARP inhibitors (e.g., talazoparib): may exploit synthetic lethality in ALT or ATRX‐deficient sarcomas; also triggers STING‐mediated immune activation. STING agonists (e.g., ADU‐S100, MK‐1454): although results have been modest in solid tumours, combination with PARPi or DNA‐damaging agents may synergise in ALT‐rich sarcomas.

### 5.2 Metabolism‐targeted combinations with ICIs

Glycolysis inhibitors (2‐DG): inhibits glucose uptake and glycolysis; in preclinical sarcoma models, reduces lactate‐mediated T‐cell suppression. LDHA inhibitors (e.g., FX11, NCI‐006): reduce lactic acid and restore effector T‐cell function; early‐stage trials are evaluating safety. Glutaminase inhibition (CB‐839 [telaglenastat]): phase I/II trials in combination with nivolumab in RCC and TNBC showed manageable toxicity. → NCT03190941: CB‐839 + nivolumab includes soft tissue sarcoma cohorts with promising preliminary immune activation. IDO1 inhibitors (Epacadostat, BMS‐986205): despite failed phase III trials in melanoma, combining IDO1 inhibition with ICIs in metabolically immunosuppressive sarcomas remains of interest. NCT04106414: BMS‐986205 + nivolumab includes rare tumours. Hypoxia and ROS modulators: hypoxia‐activated prodrugs (e.g., evofosfamide)—target HIF‐driven TME; may complement PD‐1/VEGF blockade. Mitochondrial ROS enhancers (e.g., elesclomol): induce immunogenic stress and have been explored in osteosarcoma.

### 5.3 Epigenetic and immunometabolic remodelling therapies

Given the epigenetic regulation of both telomere function and immune gene expression, epigenetic modifiers offer promising synergy with ICIs—EZH2 inhibitors (e.g., tazemetostat): approved for epithelioid sarcoma; enhances MHC‐I expression and T‐cell infiltration. NCT05023655: a clinical trial evaluating the combination of tazemetostat and atezolizumab in INI1‐deficient tumours. HDAC inhibitors (e.g., entinostat): downregulate PD‐L1 and suppress MDSCs; being studied with ICIs in sarcoma. DNMT inhibitors (e.g., azacitidine): induce viral mimicry via endogenous retroviral elements, potentially enhancing STING activation in ALT+ tumours.

### 5.4 Emerging directions and precision immunometabolic stratification

Immunometabolic profiling and biomarkers: tools such as PET tracers (e.g., 18F‐FDG, glutamine‐based tracers) and MRSI are utilised to characterise tumour metabolism and inform therapeutic strategies. Telomere FISH, TERT promoter methylation and ATRX mutation status to stratify patients into TMM categories. Multi‐omics integration (RNA‐seq + metabolomics + spatial proteomics) to predict immunotherapy response. Organoid and PDX models of sarcoma: patient‐derived sarcoma organoids with engineered TMM and metabolic profiles are being developed for drug testing. Humanised PDX models enable in vivo evaluation of immunometabolic therapies in personalised settings. Cellular therapies and oncolytic viruses: TERT‐specific CAR‐T cells and ALT‐sensitised oncolytic viruses (e.g., delta24‐RGD) are under investigation. Combination with metabolic adjuvants (e.g., metformin, dichloroacetate) may improve persistence and efficacy of adoptive cell therapies (Table 5).

**TABLE 5:** Key challenges and potential solutions for translating telomere maintenance mechanism (TMM)‒metabolism‒immunity insights into clinical practice.

Challenge	Potential solutions
Heterogeneity of TMM and metabolism within sarcoma subtypes	Dynamic biomarker monitoring, liquid biopsy for TERT/ALT signatures
Immunosuppressive TME with poor TIL infiltration	TME reprogramming via lactate blockade, arginine supplementation, or STING agonism
Lack of predictive biomarkers for ICI response	Combined metabolic, telomere and immune gene signature; AI‐driven stratification
Limited efficacy of single‐agent ICI in sarcoma	Multi‐targeted combinations: ICI + metabolic + epigenetic + TMM‐based therapies

*Note*: This table outlines major obstacles—including intratumoural heterogeneity, immunosuppressive TME, lack of predictive biomarkers and limited efficacy of ICIs in sarcomas—together with potential solutions such as biomarker‐driven stratification, TME reprogramming and rational combination therapies.

Abbreviations: ALT, alternative lengthening of telomeres; ICI, immune checkpoint inhibitor; TERT, telomerase reverse transcriptase; TME, tumour microenvironment.

Therapeutic targeting of the TMM‒metabolism‒immunity axis offers a promising route to overcome the current immunotherapy resistance in sarcomas. Future success depends on precision medicine strategies, integrating molecular profiling, functional assays and adaptive clinical trial designs to identify the right combinations for the right patients. With advances in metabolic imaging, TMM detection and immune phenotyping, the next generation of sarcoma treatments may finally unlock the potential of personalised immunotherapy.

Recent advances in artificial intelligence (AI) and machine learning (ML) offer powerful tools for integrating telomere status, metabolic signatures and immune profiling into unified predictive models. By analysing multi‐omic datasets, including genomics, transcriptomics, metabolomics and spatial proteomics, AI/ML approaches can identify biomarker combinations that stratify sarcoma patients more precisely and forecast immunotherapy response. Incorporating such computational frameworks into translational research may accelerate the development of biomarker‐driven, subtype‐specific therapeutic strategies within the TMM‒metabolism‒immunity axis.

An additional opportunity lies in the integration of metabolic imaging modalities (e.g., hyperpolarised MRI, PET tracers) with telomere profiling, which could serve as a precision medicine tool to stratify patients and guide rational combinatorial therapy in sarcomas.

### 5.5 Therapeutic implications

Before therapies directed at the telomere‒metabolism‒immunity (TMM‒metabolism‒immunity) axis can be applied to sarcomas, their practicality in the clinic and potential safety concerns must be thoroughly evaluated.^56^ Several critical considerations emerged from recent findings and were emphasised in our revised review.

#### 5.5.1 Toxicity and safety challenges

While TERT‐directed immunotherapies and metabolic inhibitors hold promise, they also raise concerns regarding on‐target/off‐tumour effects and systemic toxicity. TERT‐based vaccines and TCR‐engineered T cells may inadvertently target normal stem cells with residual telomerase activity, while metabolic inhibitors such as glutaminase or LDHA blockade can disrupt systemic homeostasis. Balancing therapeutic potency with tolerability will be essential for successful translation.

#### 5.5.2 Delivery systems

The implementation of nucleic acid‐based approaches, including siRNA or antisense oligonucleotides targeting TERT or ATRX, faces substantial barriers related to stability, biodistribution and tumour‐specific delivery. Advances in nanoparticle formulations, exosome‐mediated delivery and local administration strategies are being explored to overcome these limitations and maximise efficacy in sarcoma patients.

#### 5.5.3 Patient stratification

Given the heterogeneity of TMM status, metabolic phenotypes and immune microenvironment in sarcomas, biomarker‐guided patient selection is indispensable. Integrating telomere maintenance subtype (TERT vs. ALT), metabolic rewiring signatures (glycolysis, glutamine dependence) and immune profiling (inflamed vs. cold TME) into diagnostic frameworks may enable precision enrollment in clinical trials and improve therapeutic outcomes.^56^, ^62^

#### 5.5.4 Trial design limitations

Clinical progress has been hampered by small and histologically diverse sarcoma cohorts, making it difficult to draw definitive conclusions. Endpoint selection remains challenging, particularly in rare sarcoma subtypes where progression‐free survival and immunological correlates may not align with overall survival. To address these challenges and expedite the clinical translation of TMM‒metabolism‒immunity‐targeted strategies, adaptive trial designs and global collaborations will be essential.^63^

While telomere maintenance and metabolic reprogramming are often linked to immune escape, some studies report conflicting findings. For example, ALT‐positive sarcomas have been reported to display heterogeneous immune phenotypes, with some studies describing increased antigen presentation and T‐cell infiltration, while others indicate immune exclusion and PD‐L1 upregulation. Similarly, the effects of metabolic inhibitors appear inconsistent across sarcoma models: glutaminase blockade sensitised certain subtypes to ICIs, whereas in others it produced minimal benefit or even promoted adaptive resistance. These discrepancies highlight the complexity of telomere‒metabolism‒immunity interactions and emphasise the need for context‐specific, subtype‐tailored therapeutic strategies.^64^, ^65^, ^66^

By critically addressing toxicity, delivery, stratification and trial design, these therapeutic considerations provide a framework for translating mechanistic insights into clinically actionable strategies. Importantly, the convergence of telomere biology, metabolic reprogramming and immune modulation underscores the necessity of rationally designed, biomarker‐driven combinations to achieve durable benefit in sarcoma patients.^3^, ^67^, ^68^

### 5.6 Paediatric versus adult sarcomas: divergent TMM‒metabolism‒immunity interplay

Biological differences between paediatric and adult sarcomas shape telomere regulation, metabolic reprogramming and the immune microenvironment, thereby affecting therapeutic approaches. Paediatric sarcomas, such as osteosarcoma and rhabdomyosarcoma, often display higher proliferative indices and unique developmental signalling pathways that shape TMM activity. For instance, telomerase activation is more frequently observed in paediatric tumours, whereas ALT is enriched in certain adolescent and young adult sarcomas. These differences suggest age‐dependent variations in replicative immortality mechanisms. Distinct metabolic signatures are observed in paediatric versus adult sarcomas. Paediatric tumours demonstrate pronounced glutamine and serine dependencies to sustain rapid growth, while adult sarcomas more commonly exhibit adaptations in lipid metabolism and oxidative phosphorylation. Such metabolic rewiring not only fuels tumour progression but also differentially modulates the tumour immune microenvironment.^69^, ^70^, ^71^, ^72^, ^73^

Immune contexture further underscores the paediatric‒adult divide. Paediatric sarcomas often present with lower mutational burdens and less inflamed immune landscapes, resulting in reduced responsiveness to ICIs. In contrast, some adult sarcomas harbour higher genomic instability, generating neoantigens that may increase immunogenicity, albeit in a context‐dependent manner.

Taken together, these differences highlight the importance of age‐specific biomarker development and therapeutic tailoring. Paediatric sarcomas may benefit from strategies targeting telomerase activity and metabolic addictions, whereas adult sarcomas may be more amenable to combinatorial approaches that integrate ALT modulation, metabolic inhibitors and immunotherapy. Recognising these distinctions will be essential for designing precision interventions across diverse sarcoma populations.^66^, ^74^, ^75^, ^76^, ^77^

### 5.7 Potential resistance mechanisms and mitigation strategies

Despite encouraging advances in targeting the TMM‒metabolism‒immunity axis, resistance to combined therapies remains a significant challenge. Potential mechanisms include clonal heterogeneity, which enables subpopulations of tumour cells to evade selective pressures; metabolic plasticity, allowing tumours to rewire nutrient dependencies when one pathway is inhibited; and immune exhaustion, driven by chronic antigen exposure and inhibitory signalling. To mitigate these challenges, several strategies can be considered: dynamic biomarker‐guided monitoring (e.g., liquid biopsy for TERT/ALT status, metabolic tracers) to detect emerging resistant clones. Adaptive therapy design, alternating or layering metabolic and telomere‐targeted inhibitors with ICIs to prevent pathway compensation. Immune reinvigoration approaches, such as cytokine modulation, checkpoint blockade beyond PD‐1/CTLA‐4 and adoptive T‐cell transfer, to overcome exhaustion. Combination with epigenetic reprogramming agents, which can restore antigen presentation and sensitise resistant tumours.

Together, these strategies highlight the need for precision, multilayered interventions to overcome resistance and achieve durable responses in sarcoma patients.

## 6 RECENT CLINICAL ADVANCES AND TRIALS

Recent clinical developments have begun to validate the therapeutic potential of targeting TMMs and tumour metabolism in sarcomas, particularly in combination with ICIs. These trials are not only redefining standard care but also laying the foundation for precision immunometabolic therapy in mesenchymal malignancies.

### 6.1 Engineered T‐cell therapies targeting TMM‐linked antigens

Afamitresgene Autoleucel (afami‐cel, Tecelra) was approved by the United States. In August 2024, the FDA granted approval to afami‐cel, marking it as the first TCR‐based engineered therapy authorised for use in solid tumours. It targets the MAGE‐A4 cancer‐testis antigen, which is highly expressed in synovial sarcoma and other soft tissue sarcomas. In the SPEARHEAD‐1 trial (NCT04044768),^78^ afami‐cel demonstrated an objective response rate of ∼39%, with some durable responses in patients with unresectable or metastatic synovial sarcoma. Tumours with high TERT expression and low HLA class I loss were more likely to respond, suggesting a link between telomere dynamics and antigen presentation capacity.^79^, ^80^

### 6.2 Telomerase‐based immunotherapies

TERT‐based peptide and DNA vaccines: several peptide formulations, including GV1001, UV1 and GX301, are undergoing evaluation at different phases of clinical development. UV1, a synthetic long peptide vaccine targeting hTERT, is being evaluated in multiple solid tumours in combination with PD‐1 blockade. In a phase II trial (NCT03715946) in melanoma, UV1 + pembrolizumab showed enhanced T‐cell infiltration and improved survival. Extension into sarcoma is under discussion, particularly for TERT‐high tumours like Ewing sarcoma and rhabdomyosarcoma. Adoptive T‐cell therapies: trials using TERT‐specific TCR‐engineered T cells are in early‐phase development, showing preclinical efficacy in TERT‐overexpressing sarcoma cell lines. Combination with STING agonists or hypomethylating agents may potentiate antigen presentation and overcome immune exhaustion.^81^, ^82^, ^83^, ^84^, ^85^, ^86^, ^87^, ^88^

### 6.3 Metabolic modulation to boost immune response

ADI‐PEG 20 (pegylated arginine deiminase): many sarcomas, including myxofibrosarcoma and leiomyosarcoma, are arginine auxotrophs due to ASS1 deficiency. In a phase 1b/2 clinical trial (NCT03254732) testing ADI‐PEG 20 in combination with pembrolizumab, more than 40% of patients with advanced sarcoma experienced disease control, with several individuals showing durable partial responses.^89^ This suggests that arginine depletion reshapes the TME and enhances ICI sensitivity by modulating myeloid suppressor cells and T‐cell metabolism. Glutaminase inhibitors: the ENLIGHTEN trial (NCT02771626) is assessing telaglenastat (CB‐839)^90^ in combination with immune checkpoint blockade, with inclusion of patients who have rare solid tumours. Sarcomas with elevated MYC signalling or mTOR activation may benefit from glutamine‐targeted strategies, especially when T‐cell exhaustion is driven by nutrient competition. LDHA inhibitors^91^ and lactate blockers^92^, ^93^: preclinical models in UPS and dedifferentiated liposarcoma suggest that lactate^39^ inhibition restores CD8+ T‐cell function and enhances ICI efficacy. Several compounds, including FX11^94^ and GNE‐140, are being developed for solid tumours and may be repurposed for sarcoma in upcoming trials.

### 6.4 Oncolytic virus therapy in TMM‐disrupted sarcomas

Engineered from vaccinia, Pexastimogene Devacirepvec (Pexa‐Vec)^95^ is designed to preferentially propagate within tumour tissue and to induce antitumour immunity by engaging the TLR and STING signalling cascades. In early‐phase trials, Pexa‐Vec^96^ has been shown to increase TIL infiltration and cytokine release, acting as a priming agent for checkpoint blockade. Current trials (e.g., NCT03071883) are assessing Pexa‐Vec + nivolumab in soft tissue sarcoma, with a focus on ALT+ or immune‐excluded tumours. DNX‐2401^97^ (Delta‐24‐RGD): originally developed for glioma, now under exploration in sarcomas, especially those with defective DNA repair (e.g., ATRX/TP53 mutations), as in ALT+ osteosarcoma. OVs can induce immunogenic cell death, enhance neoantigen presentation, and be further potentiated by TMM‐targeting agents.^98^, ^99^, ^100^

### 6.5 Epigenetic and immunometabolic combination trials

The EZH2 inhibitor tazemetostat, authorised by the FDA for INI1‐deficient epithelioid sarcoma, demonstrates clinical benefit and may enhance PD‐L1 blockade through its effects on chromatin remodelling and induction of immune‐related gene expression. In bone and soft tissue sarcomas, entinostat—an HDAC inhibitor^101^, ^102^—is under clinical evaluation in the NCT04308330 trial, where it is administered together with nivolumab and ipilimumab.^103^ Preliminary data show reversal of immune exclusion and reprogramming of myeloid cells. Simultaneous targeting of DNMT and HDAC can expose endogenous retroviral elements (ERVs)^104^ triggering viral mimicry pathways—an effect that is especially pertinent in ALT‐positive sarcomas characterised by chromatin instability (Table 6).^105^

**TABLE 6:** Summary of promising trials targeting telomere maintenance mechanisms and metabolism in sarcoma.

Therapeutic agent	Target	Trial ID	Tumour type(s)	Status/notes
Afami‐cel	MAGE‐A4	NCT04044768	Synovial sarcoma	FDA approved (2024)
UV1 vaccine	hTERT	NCT03715946	Melanoma, potential in sarcoma	Phase II
ADI‐PEG20 + pembrolizumab	Arginine metabolism	NCT03254732	Soft tissue sarcoma	Phase 1b/2, >40% DCR
Pexa‐Vec + ICI	Oncolytic virus	NCT03071883	Solid tumours including STS	Early phase
C8‐839 + nivolumab	Glutaminolysis	NCT02771626	Various solid tumours	Ongoing
Tazemetostat	EZH2	NCT05023655	Epithelioid sarcoma	FDA approved
Entinostat + ICI	Epigenetic reprogramming	NCT04308330	Sarcoma	Recruiting

Abbreviation: DCR, disease control rate; ICI, immune checkpoint inhibitor; STS, soft tissue sarcoma.

Representative early‐ and late‐phase clinical trials are listed with therapeutic agents, targets, trial IDs, tumour types and clinical status, encompassing engineered TCR therapies, telomerase‐based vaccines, metabolic modulators, oncolytic viruses and epigenetic combinations.

These recent clinical advances highlight a growing recognition of the TMM‒metabolism‒immune axis in sarcoma biology and therapy. Integration of TMM profiling (e.g., TERT expression, ALT status) with metabolic and immune biomarkers will be essential to guide biomarker‐driven combination therapies. The future of sarcoma immunotherapy lies in personalised approaches that simultaneously target telomere dynamics, metabolic vulnerabilities and immune checkpoints, unlocking synergistic responses and overcoming the intrinsic resistance that has historically limited progress in this challenging malignancy.^106^

## 7 CONCLUSIONS AND FUTURE DIRECTIONS

Sarcomas represent a highly heterogeneous group of malignancies that have historically shown limited responsiveness to ICIs. As efforts to expand the reach of immunotherapy continue, it is becoming increasingly clear that tumour‐intrinsic features—particularly TMMs and metabolic reprogramming—play pivotal roles in shaping the immunological landscape of these tumours. This review has highlighted the dual and often interdependent influence of TMMs and metabolic pathways in modulating immune surveillance and therapy response.

Telomerase activation and ALT not only enable limitless replicative potential but also intersect with immune escape mechanisms. For example, TERT upregulation can induce immune checkpoint expression and dampen cytotoxic immune responses, while ALT‐driven genomic instability may paradoxically enhance tumour immunogenicity yet simultaneously desensitise innate immune sensing pathways such as cGAS‒STING. Metabolic rewiring in sarcomas—characterised by glutamine addiction, arginine auxotrophy, aerobic glycolysis and FAO—reshapes the TME by creating metabolic competition and suppressive niches that impair T‐cell function and antigen presentation. Notably, many of these metabolic adaptations are influenced by or directly impact telomere regulatory networks, forming a unified axis of immunomodulation.

Encouragingly, recent clinical advances have begun to exploit this axis. Integrated therapeutic strategies are strongly supported by recent advances, including the FDA's approval of afamitresgene autoleucel (Tecelra) for synovial sarcoma, encouraging outcomes from telomerase‐targeted vaccines, and early‐phase evidence showing benefit of metabolic modulators combined with ICIs. Oncolytic virus therapies, epigenetic drugs and emerging agents targeting TMM‒metabolism crosstalk further enrich the therapeutic arsenal.^107^

However, several critical challenges remain. First, reliable biomarkers that reflect the dynamic interplay between TMM status, metabolic phenotype and immune competence are urgently needed. Second, sarcomas exhibit considerable intra‐ and inter‐subtype heterogeneity, necessitating personalised treatment paradigms. Third, the development of rational combination regimens—such as TERT vaccines^108^ with glutaminase inhibitors and checkpoint blockade—must be guided by robust mechanistic insights and supported by adaptive clinical trial designs. In addition, translational hurdles—including toxicity management, delivery system optimisation and appropriate patient selection—must be addressed to ensure safe and effective implementation.^109^

Looking ahead, future research should focus on: (1) molecular stratification of sarcoma patients^56^ based on TMM signatures (e.g., TERT‐high vs. ALT+) to facilitate the precise selection of patients for clinical trials. (2) Development of diagnostic and predictive biomarkers that integrate telomere status, metabolic rewiring and immune profiling to guide therapy.^110^ (3) Preclinical model systems that faithfully recapitulate the complex telomere‒metabolism‒immunity interplay, enabling mechanistic dissection and therapeutic testing. (4) Identification of unmet clinical needs, including tools for early detection of TMM activity, biomarkers for therapy resistance, and strategies to overcome delivery and toxicity challenges. (5) Design of rational combination regimens that synergise TMM targeting, metabolic modulation and immune activation, supported by biomarker‐driven and adaptive clinical trial frameworks.^111^

In conclusion, by embracing the intricate crosstalk between telomere biology, cellular metabolism and immune regulation, the field is poised to unlock new dimensions in sarcoma therapy. A deeper understanding of these relationships will not only illuminate the underpinnings of immune resistance but also pave the way for innovative, multi‐pronged, and clinically actionable treatment strategies capable of transforming outcomes for patients with these challenging malignancies.

## AUTHOR CONTRIBUTIONS

*Conceptualisation, methodology, formal analysis, writing—original draft, writing—review and editing and supervision*: Ji‐Yong Sung. *Funding acquisition*: June Hyuk Kim. Both the authors have read and agreed to the published version of the manuscript.

## CONFLICT OF INTEREST STATEMENT

The authors declare they have no conflicts of interest.

## ETHICS STATEMENT

N/A

## Data Availability

Data sharing not applicable to this article as no datasets were generated or analysed during the current study.
